# Natural Immunity to *Klebsiella pneumoniae* in Mothers and Their Infants in Malawi

**DOI:** 10.1093/ofid/ofag676

**Published:** 2026-10-22

**Authors:** Nathalie Karaky, Oliver Pearse, Robert Lawrence, Emma Bownes, Edward Hildebrand, Allan Zuza, Luis Gadama, David Lissauer, Samantha Lissauer, Kondwani Kawaza, Nicholas Feasey, David Goldblatt

**Affiliations:** Great Ormond Street Institute of Child Health, University College London, London, UK; Department of Clinical Sciences, Liverpool School of Tropical Medicine, Liverpool, UK; Malawi-Liverpool-Wellcome Programme, Kamuzu University of Health Sciences, Blantyre, Malawi; Great Ormond Street Institute of Child Health, University College London, London, UK; Great Ormond Street Institute of Child Health, University College London, London, UK; Great Ormond Street Institute of Child Health, University College London, London, UK; Malawi-Liverpool-Wellcome Programme, Kamuzu University of Health Sciences, Blantyre, Malawi; The School of Medicine, University of St. Andrews, St. Andrews, UK; Kamuzu University of Health Sciences, Blantyre, Malawi; Institute of Life Course & Medical Sciences, Faculty of Health Sciences, University of Liverpool, Liverpool, UK; Malawi-Liverpool-Wellcome Programme, Kamuzu University of Health Sciences, Blantyre, Malawi; University of Liverpool Institute of Infection and Global Health, Liverpool, UK; Malawi-Liverpool-Wellcome Programme, Kamuzu University of Health Sciences, Blantyre, Malawi; Kamuzu University of Health Sciences, Blantyre, Malawi; Department of Clinical Sciences, Liverpool School of Tropical Medicine, Liverpool, UK; Malawi-Liverpool-Wellcome Programme, Kamuzu University of Health Sciences, Blantyre, Malawi; The School of Medicine, University of St. Andrews, St. Andrews, UK; Great Ormond Street Institute of Child Health, University College London, London, UK

**Keywords:** antibody titers, *Klebsiella pneumoniae*, maternal vaccination, neonatal sepsis, O antigen, sero-epidemiology

## Abstract

**Background:**

*Klebsiella pneumoniae* is a leading cause of neonatal sepsis in low- and middle-income countries, with high associated mortality. Anti–*K. pneumoniae* maternal antibodies may provide early-life protection to neonates, yet data about their prevalence, transplacental transfer, and functional activity remain limited.

**Methods:**

We conducted a study in Malawian mother–infant dyads measuring immunoglobulin G (IgG) to *K. pneumoniae* O1 serotypes, O1αβ,2α and O1αβ,2β, and functional immunity. Immunity was also analyzed in dyad sera from (i) culture-confirmed *K. pneumoniae* infection (cases), (ii) non–*K. pneumoniae* infections (sick controls), and (iii) healthy controls.

**Results:**

Anti O1-IgG titers were similar in all mothers and significantly greater in infants. Similarly, functional immunity was significantly higher in mothers than their infants. Transfer ratios for functional immunity between mother and infant were significantly lower (0.017) than those seen for anti-O1 IgG (0.8). While IgG to O1αβ,2α and O1αβ,2β and functional immunity were significantly correlated in healthy infants, this was not the case in maternal serum. Neither *Klebsiella*-specific maternal IgM titers nor IgG subclasses could explain this; thus additional factors, in addition to anti-O1 IgG, are likely contributing to functional immunity.

**Conclusions:**

Natural anti O1 antibodies are ubiquitous in healthy mothers in Malawi, and IgG is efficiently transferred to neonates. Reduced functional immunity in infants compared with mothers, as demonstrated in this study, may underpin the susceptibility to *K. pneumoniae* infection in early life, justifying the exploration of maternal immunization to prevent early-life infection. Further studies are needed to define the characteristics of anti–*K. pneumoniae* immunity that might be associated with clinical protection.

*Klebsiella pneumoniae* is a significant opportunistic pathogen and a major cause of health care–associated infections (HAIs). It has been identified as one of the primary causes of neonatal sepsis, accounting for >20% of cases in low- and middle-income countries (LMICs), where 99% of global neonatal mortality occurs [1–5]. The burden of infections caused by *K. pneumoniae* is exacerbated by its strong association with antimicrobial resistance (AMR). It was estimated to be responsible for 19.4% of AMR-attributable deaths in children under 5 years of age in 2019 in South Asia, Bangladesh, and Africa [3]. Isolates of *K. pneumoniae* are often resistant to key antibiotics in the treatment of neonatal sepsis, such as first- and second-line therapies, aminopenicillins, gentamicin, and third-generation cephalosporins, rendering infections difficult or impossible to treat with locally available antibiotics [6]. *K. pneumoniae* has therefore been listed by the World Health Organization (WHO) as an AMR priority pathogen [7]. The high burden of disease and treatment failure associated with AMR have shifted focus during the past decade toward approaches to control and prevent infection, including the development of vaccines, which in the case of neonatal sepsis would need to be maternally administered [8].

Protective immunity against symptomatic *K. pneumoniae* infection is largely mediated by the humoral arm of the immune system; antibodies target key surface antigens including the capsular polysaccharide (K antigen) and the O-specific polysaccharide of lipopolysaccharide (O antigen). The K antigen is a major virulence determinant, conferring resistance to phagocytosis and complement-mediated killing [9] and, as for other encapsulated bacteria, is a target for functional antibodies. Antibodies against O antigens, particularly anti-O1 and anti-O2, derived from human memory B cells, have also been shown to mediate complement activation and promote bacterial clearance in murine models [10]. Clinical studies have shown elevated immunoglobulin G (IgG) responses to O antigens in convalescent serum, but the extent to which such antibodies and anti-K antibodies confer protection against disease in humans is unknown [11, 12]. Despite this, both K and O antigens are putative vaccine targets, although their diversity remains a potential barrier, with >160 K types and 14 O types described [12, 13].

Studies of natural immunity to *K. pneumoniae* in humans could aid in the understanding of immunity likely to be protective and consequently guide vaccine development. O1 is the single most prevalent antigen, and its 2 predominant subtypes (O1αβ,2α, O1αβ,2β) account for ∼50% of neonatal infections [13]. It is thus a sensible target for studying the seroprevalence of *K. pneumoniae* O antigen–specific maternal IgG, the kinetics of the transfer of such antibodies to the newborn infant, and the possible relationship to susceptibility to infection.

The number of published human studies specifically evaluating antibody levels against *K. pneumoniae* in mothers and/or neonates is limited, with only 2 in the peer-reviewed literature [14, 15]. A case–control study conducted in Botswana identified 8 cases and evaluated 32 controls and found that higher maternal and neonatal anti–*K. pneumoniae* IgG was associated with reduced risk of neonatal sepsis, but IgG was measured to a whole bacterial isolate representing a single invasive strain, so little can be deduced about the putative protective target of IgG [14]. A twin cohort study measured IgG to purified LPS from a single strain of *Klebsiella* and showed that there was a positive correlation between maternal antibody levels and those observed in newborns. Transplacental transfer of maternal total IgG and anti-LPS *Pseudomonas* IgG antibodies was significantly lower in newborns born before 34 weeks of gestational age [15].

Here, we characterize naturally acquired humoral immunity to *K. pneumoniae* in mothers and their neonates in Malawi, with a specific focus on antibodies directed against predominant O antigens. By defining antibody prevalence and patterns of maternal-to-neonatal transfer in a high-burden setting, we add to the understanding of early-life immunity to *K. pneumoniae*.

## METHODS

### Study Design and Participants

This study was nested within a prospective cohort study conducted at Queen Elizabeth Central Hospital (QECH) in Blantyre, Malawi, from April 2021 to April 2023. Mother and infant pairs (henceforth termed “dyads”) were recruited on the labor ward if they were expected to deliver at QECH or recruited if <3 months old from the pediatric wards. Dyads were excluded if the mother declined to give consent, was under 16 years old, or did not speak either English or Chichewa. Dyads were followed up to 3 months postdelivery to record outcomes. Clinical data were collected, encrypted, and anonymized.

### Case and Control Selection

Infants were categorized into 3 different groups: *K. pneumoniae* neonatal sepsis cases (henceforth “cases”), healthy controls, and sick controls. Cases were defined as neonates/infants <3 months old who had *K. pneumoniae* isolated from their bloodstream or cerebrospinal fluid (CSF). Sick controls were neonates/infants <3 months old who met the clinical criteria for infection (documented fever ≥38 °C, clinical diagnosis of sepsis, or meningitis as per the clinical team), and/or had a positive blood culture or CSF for an organism that was not *K. pneumoniae* (Supplementary Table 1). Healthy controls, recruited from the labor or pediatric ward, were infants who did not meet the above criteria for sepsis or meningitis by the final follow-up at 3 months. Sick and healthy controls were selected to achieve an approximate case–control ratio of 2:1 and 4:1, respectively. Controls were also matched to cases by gestational age to account for the time-dependent transfer of maternal antibodies. Over 30% of maternal healthy controls were intentionally selected based on enrichment for *K. pneumoniae* stool carriage, under the premise that their infants had been exposed to *K. pneumoniae* without subsequently developing an infection.

### Sample Processing and Data Collection

Maternal serum, cord blood, or neonatal serum and a stool sample or 2 rectal swabs were collected from all participants. *K. pneumoniae* in stool samples and rectal swabs was identified using melt-curve polymerase chain reaction analysis, as previously described [16]. Blood and CSF samples of infants presenting with clinical signs of neonatal sepsis were handled as previously described [17, 18].

### Whole-Genome Sequencing and Analysis for Strain Typing

Samples underwent whole-genome sequencing (WGS) at the Wellcome Sanger Institute at 364 plex on the Novaseq using an SP flowcell generating 150-bp paired-end reads, as previously described (Supplementary Methods) [17]. O typing, K typing, and sequence typing (ST) were performed using Kleborate, version 3.0.0 [19].

### Binding Antibody Titers

Total IgG specific to *K. pneumoniae* O1αβ,2α and O1αβ,2β antigens were quantified using a multiplexed bead-based immunoassay, performed as previously described [20]. In addition, the assay was adapted to measure IgG1, IgG2, IgG3, and IgM specific to O1αβ,2α using isotype and subclass-specific secondary antibodies.

### Pneumococcal Antigen Titers

A multiplex electrochemiluminescence (ECL) immunoassay (MSD, Rockville, MD, USA) was used to measure natural antibodies (IgG) to 9 capsular *Streptococcus pneumoniae* antigens (5, 6A, 6B, 7F, 9V, 11A, 14, 19A, 19F), as previously described by us [21].

### Opsonophagocytic Killing Assay

An opsonophagocytic killing assay (OPA) established in our laboratory was used to measure functional immunity in serum to *Klebsiella* strains expressing O1αβ [20]. Briefly, following the incubation of sera overnight with live *K. pneumoniae,* a complement source, and HL-60 cells, the number of surviving colony-forming units (CFU) were counted. The opsonic index (OI) was defined as the reciprocal of the dilution of sera required to kill 50% of the bacteria.

### Statistical Analysis

Data were analyzed using Graph Pad Prism Software, version 10.0.0 (Boston, MA, USA). Categorical clinical characteristics were compared across the 3 analysis groups using the chi-square test. When any expected cell count was <5, the Fisher exact test was used instead. Continuous clinical characteristics were compared using the Kruskal-Wallis rank-sum test. Comparisons of the geometric means of IgG titers and opsonic indices between the different groups were made using the Mann-Whitney *U* test, and Spearman's correlations were used to assess associations between the antibody titers and continuous variables. IgG subclass titers were log-transformed to approximate normal distribution and compared using an unpaired *t* test. Pearson's correlations were applied to examine associations between subclass titers and opsonic indices. Placental transfer ratios of all antibodies were defined as the ratio of infant titer/maternal titer. Transfer ratios for total IgG *Klebsiella* and pneumococcal antibodies were square-root-transformed to approximate a normal distribution. An unpaired *t* test was applied for comparisons of all transfer ratios across different groups. Statistical significance was set at *P* < .05.

## RESULTS

### Clinical Characteristics of the Cohort

A total of 631 pregnant women were recruited antenatally. However, none of the infants born to these mothers subsequently developed *K. pneumoniae* sepsis. Seventy-three of these mother–infant pairs were matched for gestational age (GA) and had sufficient volumes of samples to act as matched healthy controls. Two additional GA-matched healthy controls were recruited on day 1 and day 32 of life, respectively. Twenty-four infants with culture-confirmed *K. pneumoniae* sepsis were recruited, but only 17 had sufficient infant serum volumes to be included in the immunological analyses. Thirty-six sick control infants (without *K. pneumoniae* infection) were recruited from the pediatric wards. The clinical characteristics of each group are summarized in Table 1.

**Table 1.** Clinical Characteristics of the Overall Cohort and the Different Analysis Groups

Characteristic	Case n = 17	Healthy Control n = 74	Sick Control n = 36	*P* Value^a^
Gender, No. (%)	…	…	…	.4
Female	8 (47)	40 (54)	15 (42)	…
Male	6 (35)	34 (46)	21 (58)	…
Unknown	3 (18)	0	0	…
Outcome, No. (%)	…	…	…	.003
Alive	12 (70)	71 (96)	30 (83)	…
Dead	5 (30)	3 (4)	6 (17)	…
Location of birth, No. (%)	…	…	…	<.001
Other hospital	4 (24)	1 (1)	6 (17)	…
Other location	3 (18)	0	16 (44)	…
QECH	10 (58)	73 (99)	14 (39)	…
HIV status, No. (%)	…	…	…	.4
Negative	14 (82)	58 (78)	26 (72)	…
Positive	2 (12)	14 (19)	10 (28)	…
Unknown	1 (6)	2 (3)	0	…
Age, d Median (range), d	8.0 (3–93)	0 (0–32)	8.0 (2–87)	<.0001*
Unknown	0	0	0	…
Gestational age, wk GM (95% CI)	34.4 (32.1–36.8)	35.7 (34.5–36.4)	35.4 (33.7–37.1)	.8*
Unknown	2	0	8	…
Mode of delivery, No. (%)	…	…	…	.3
Cesarean	4 (23)	17 (23)	6 (17)	…
Vaginal	12 (71)	57 (77)	30 (83)	…
Unknown	1 (6)	0	0	…
Birthweight, mean (95% CI), g	2127 (1729–2525)	2712 (2573–2852)	2533 (2246–2819)	.007*
Unknown	1	5	4	…
Clinical sepsis, No. (%)	17 (100)	0 (0)	36 (100)	.001
Maternal Kpn colonization	3 (18)	14 (19)	3 (8.3)	.011
Neonatal Kpn colonization	7 (41)	3 (4)	11 (31)	<.001

Abbreviations: GM, geometric mean; Kpn, *Klebsiella pneumoniae*.

^a^Pearson's chi-square test; Fisher exact test; Kruskal-Wallis rank-sum test.

^*^Mann-Whitney test.

The mean GA of the cases was 34.4 weeks (95% CI, 32.1–36.8), sick controls 35.4 weeks (95% CI, 33.7–37.1), and matched healthy controls 35.6 weeks (95% CI, 34.5–36.4; *P* > .8). Mean age in days at recruitment differed significantly, with the healthy controls sampled at day 0 (range, 0–32 days) and cases/sick controls on day 8 (range, 3–93 and 2–87, respectively; *P* < .0001). Neonatal mortality was highest in the cases (30%), followed by sick controls (17.0%) and healthy controls (4%; *P* < .001). Maternal HIV prevalence did not differ significantly between the groups (*P* = .4) (Table 1).

The 17 *K. pneumoniae* isolates causing infant infections represented 12 sequence types (STs), 10 K-loci, and 5 O-loci (Supplementary Table 2). The 2 most frequently occurring O-loci were O1αβ,2β (n = 6, 35%) and O1αβ,2α (n = 4, 24%). The K-loci present in the study included capsule types KL62 (n = 3, 17%), KL2 (n = 2, 12%), KL25 (n = 2, 12%), and KL102 (n = 1, 6%). Of the 36 sick controls, 14 had positive blood cultures, which included *Escherichia coli* (n = 4), *Acinetobacter baumannii* (n = 4), *Enterococcus faecalis* (n = 2), and *Enterobacter cloacae* (n = 2). Seven blood cultures yielded bacteria deemed to be contaminants, and the rest were culture negative (Supplementary Table 1).

### IgG Specific to O1αβ,2α and O1αβ,2β in Maternal and Infant Serum Samples

Antigen-specific IgG titers in maternal sera did not differ significantly between the mothers, but all were significantly greater than their corresponding infants for both O antigens tested (O1αβ,2α, *P* < .0001; and O1αβ,2β, *P* = .0002) (Figure 1A and B). Antibody titers in infants were positively correlated with gestational age, and this was reflected in transfer ratios, which increased with gestational age, although this did not achieve statistical significance (Figure 2A). For infants where blood was only obtained in the postnatal period (cases and sick controls), transfer ratios were negatively correlated with postnatal age at recruitment (cases, *r* = –0.4, *P* = .16 for O1αβ,2α and O1αβ,2β; and sick controls, *r* = −0.4, *P* = .02 for O1αβ,2α and *P* = .007 for O1αβ,2β) (Figure 2B). Mother:infant transfer ratios (TRs) of anti-O1 IgG were significantly lower for the cases and sick controls (who were bled postnatally at recruitment) compared with healthy controls (Table 2). TRs to an antigen unrelated to anti–*K. pneumoniae* immunity (*S. pneumoniae* serotype–specific concentrations) showed the same phenomenon (Table 2). The observed reduced ratios are therefore most likely due to the differences in age at the time of sampling as IgG levels in the newborn drop rapidly after birth.

**Figure 1.**
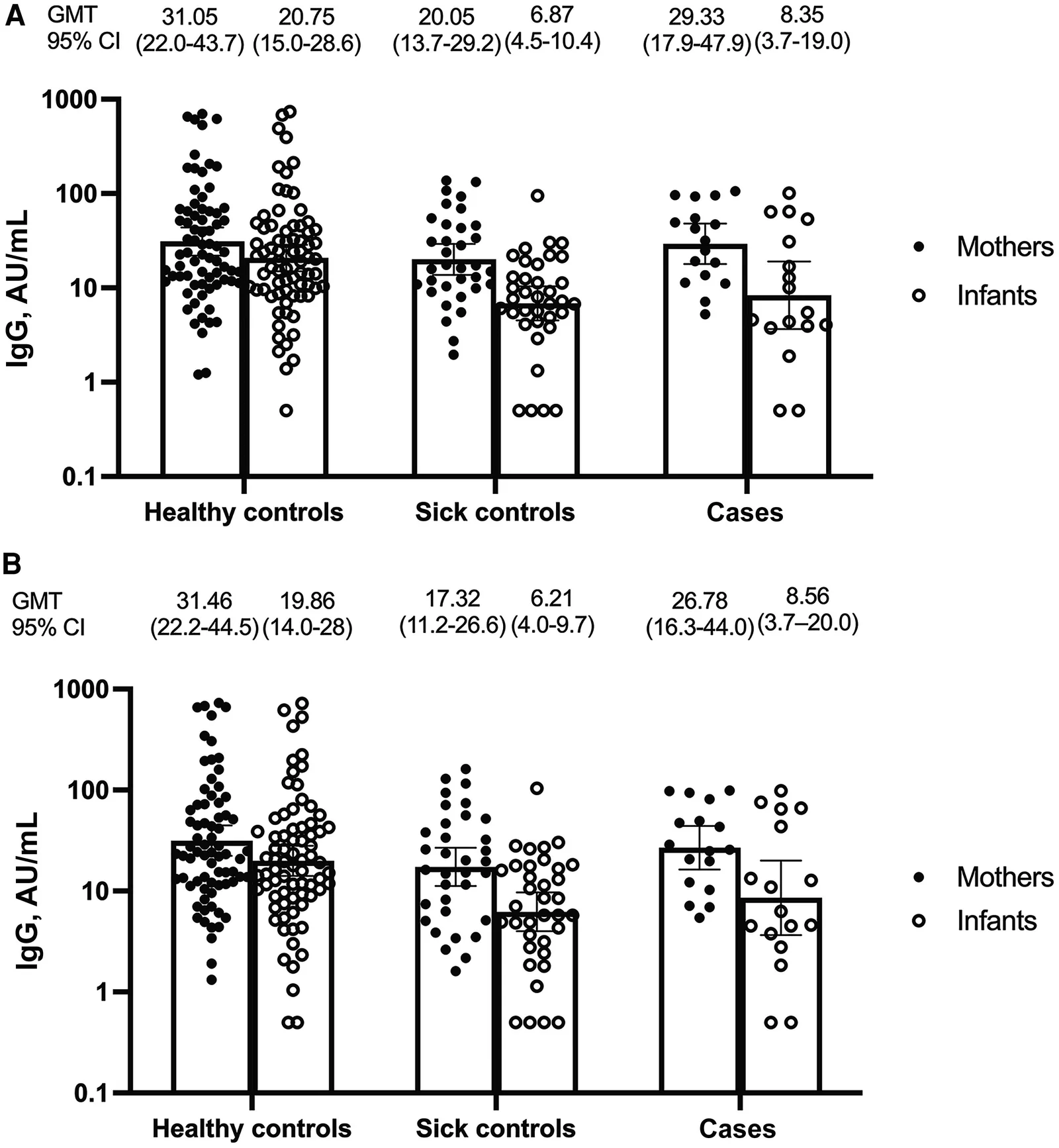
IgG titers to O1αβ,2α (A) and O1αβ,2β (B) in the sera of mothers and their infants according to whether they were classified as healthy controls (n = 73), sick controls (n = 36), or cases (n = 17). Geometric mean titers (95% CI) for each group are included on the top of each bar. Maternal titers were greater than infant titers for all 3 groups (O1αβ,2α, *P* < .0001; and O1αβ,2β, *P* = .0002), but no significant differences were found between maternal groups (*P* > .05). Differences in infant titers were significant between healthy controls and sick controls for both antigens (*P* < .0001 and *P* = .0001, respectively) and additionally between healthy controls and infant cases for anti-O1αβ,2α (*P* = .047). Group comparisons were performed using the Mann-Whitney *U* test, with *P* values indicated for statistically significant differences. Abbreviations: GMT, geometric mean titers; IgG, immunoglobulin G.

**Figure 2.**
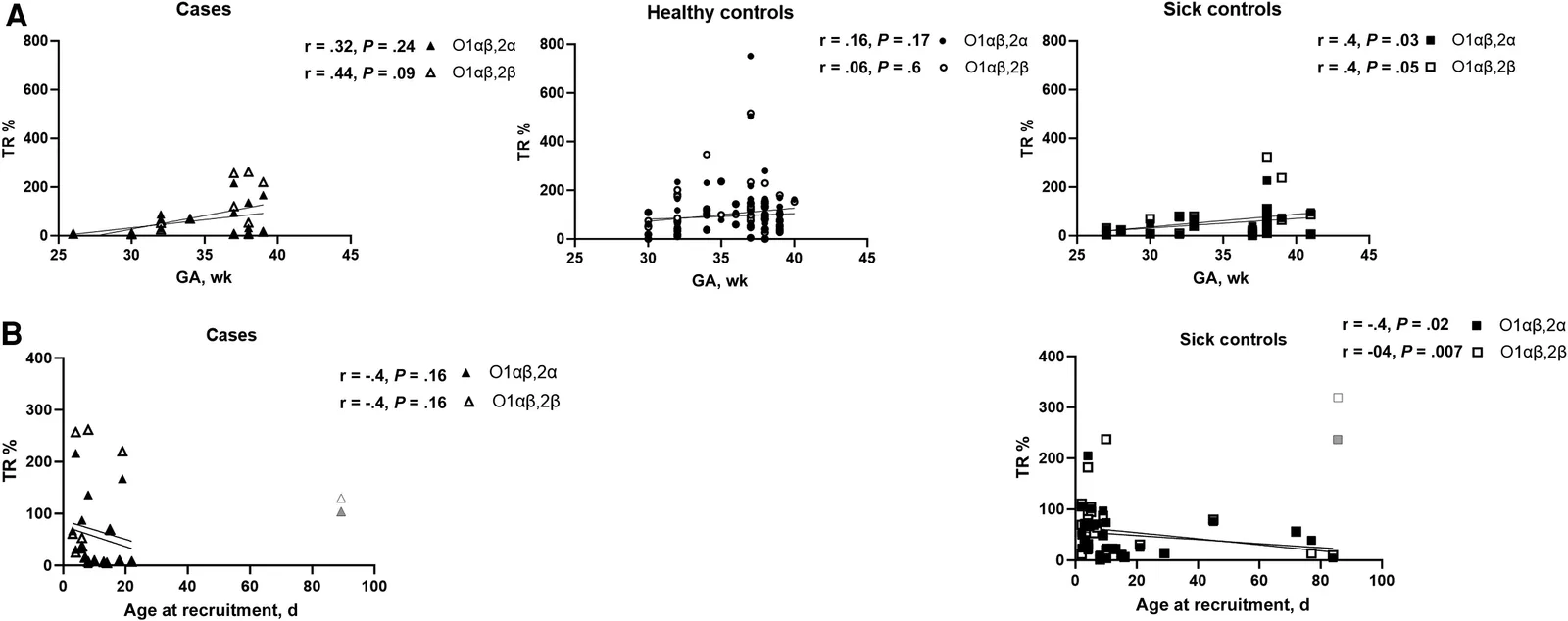
The transfer ratio of anti-O1αβ,2α and O1αβ,2β IgG as (A) a function of gestational age (in weeks) and (B) neonatal age at recruitment (in days). Healthy controls are not shown in (B) as all but 2 infants were recruited at birth. Statistical analysis performed on data excluded outliers (in gray). Abbreviation: TR, transfer ratio.

**Table 2.** Infant/Maternal Antibody Transfer Ratios for *K. pneumoniae* and *S. pneumoniae* Serotype-Specific IgG Antibodies

Serotype	Transfer Ratio Geometric Mean (95% CI)			
	Cases	Healthy Controls	Sick Controls	*P* Value
*K. pneumoniae* O1αβ,2α total IgG	0.28^*^ (0.14–0.57)	0.67 (0.50–0.88)	0.35** (0.24–0.52)	.01* .002**
*K. pneumoniae* O1αβ,2β total IgG	0.32 (0.16–0.65)	0.63 (0.48–0.82)	0.36* (0.24–0.56)	.02*
*K. pneumoniae* O1αβ,2α IgG1	NA	0.70 (0.55–0.88)	NA	---
*K. pneumoniae* O1αβ,2α IgG2	NA	0.55 (0.38–0.78)	NA	---
*K. pneumoniae* O1αβ,2α IgG3	NA	0.52 (0.34–0.80)	NA	---
*S. pneumoniae* serotype 5	0.22 (0.10–0.48)	0.42 (0.34–0.51)	0.26 (0.18–0.37)	…
*S. pneumoniae* serotype 6A	0.19 (0.07–0.49)	0.35 (0.28–0.44)	0.22* (0.16–0.30)	.01*
*S. pneumoniae* serotype 6B	0.27 (0.15–0.49)	0.39 (0.31–0.49)	0.23* (0.17–0.32)	.01*
*S. pneumoniae* serotype 7F	0.36 (0.11–1.11)	0.32 (0.25–0.39)	0.22 (0.14–0.35)	…
*S. pneumoniae* serotype 9V	0.25 (0.09–0.67)	0.42 (0.35–0.50)	0.26* (0.18–0.37)	.05*
*S. pneumoniae* serotype 11A	0.22 (0.09–0.49)	0.37 (0.31–0.45)	0.20* (0.13–0.30)	.01*
*S. pneumoniae* serotype 14	0.30 (0.17–0.55)	0.56 (0.46–0.69)	0.33 (0.21–0.51)	…
*S. pneumoniae* serotype 19A	0.23 (0.12–0.46)	0.45 (0.37–0.56)	0.31 (0.21–0.46)	…
*S. pneumoniae* serotype 19F	0.24 (0.10–0.52)	0.39 (0.31–0.49)	0.30 (0.20–0.44)	…

Statistical significance was determined using an unpaired *t* test; *P* < .05 compared with healthy controls. Data were normalized. Comparisons of TR of cases vs sick controls were not significant (*P* > .05).

Abbreviations: IgG, immunoglobulin G; TR, transfer ratio.

* or ** refers to which comparison (cases or sick controls) is significant when compared to healthy controls.

Infant cases and sick controls had anti-O1αβ,2α titers that were significantly lower than those of the healthy control infants (8.35; 95% CI, 3.66–19.09; *P* = .049; and 6.87 AU/mL; 95% CI, 4.57–10.41; *P* < .0001, respectively; vs 20.54 AU/mL; 95% CI, 14.95–28.23). Anti-O1αβ,2β titers were similar between cases and sick controls and lower than those seen in healthy controls, but only significantly lower for sick controls (8.56 AU/mL; 95% CI, 3.7–20.05; *P* = .07; and 6.21 AU/mL; 95% CI, 4.00–9.66; *P* = .0001; vs 19.58 AU/mL; 95% CI, 13.94–27.51) (Figure 1B). Titers to O1αβ,2α and O1αβ,2β were highly correlated in all samples (*r* = 0.98; *P* < .0001) (Supplementary Figure 1).

### Functional Immunity to *K. pneumoniae*

The opsonic indices for all mothers were significantly higher compared with their infants (Figure 3). Maternal-to-infant TRs for functional immunity were significantly lower (0.02–0.13) compared with binding IgG (0.28–0.67) (Supplementary Table 3). While anti-O1αβ,2α OI correlated well with IgG in the control infants (*r* = 0.41; *P* = .0003), this correlation was lost in maternal control serum (*r* = −0.05; *P* = .61) (Supplementary Figure 2).

**Figure 3.**
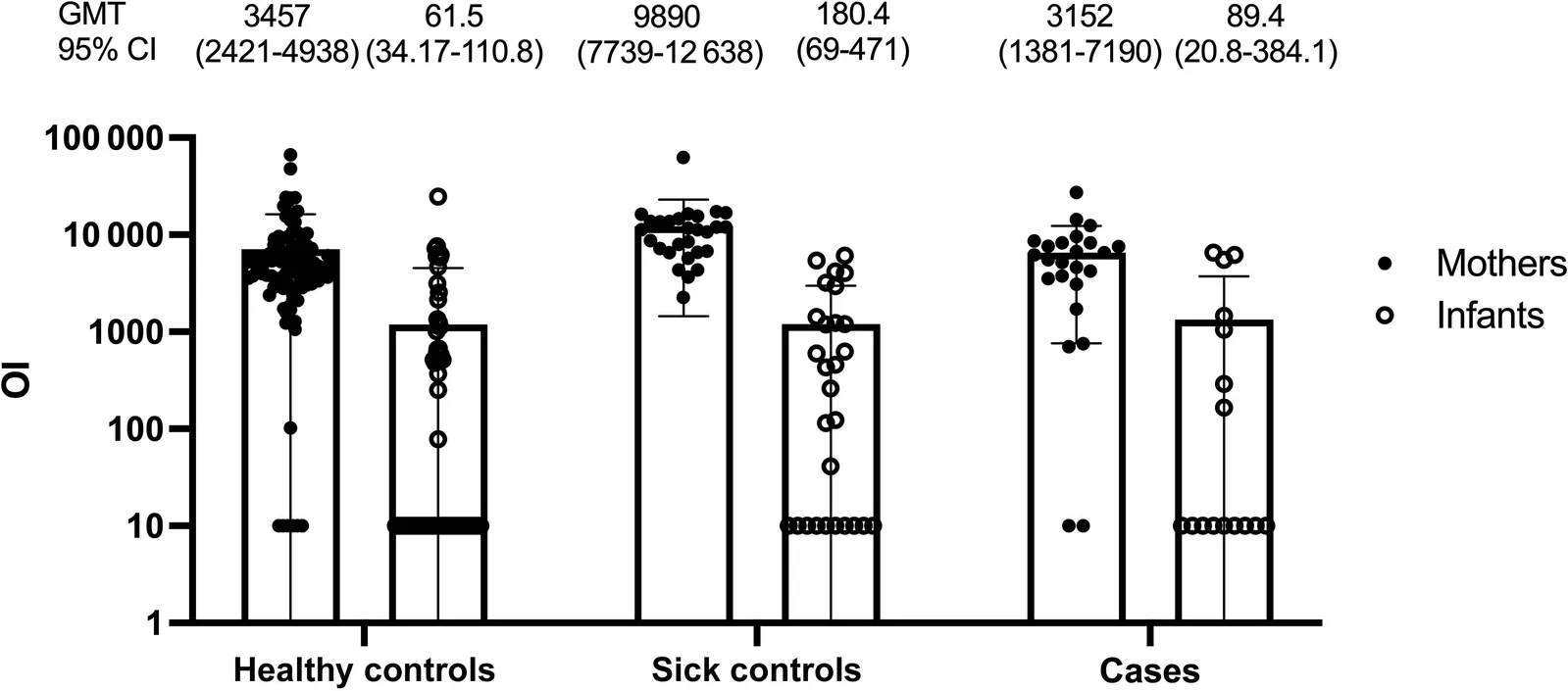
Opsonophagocytic activity against a *Klebsiella* O1αβ,2α in maternal and infant sera. Geometric mean titers (95% CI) are included on the top of each bar. Maternal OPA results for O1αβ,2α were significantly higher than corresponding neonatal values across all groups (*P* ≤ .001). Mothers of sick controls had significantly higher OI titers than mothers of healthy controls or cases (*P* = .009 and *P* < .0001, respectively). The OI of sick control infants was significantly higher than the healthy controls (*P* = .04). Group comparisons were performed using the Mann-Whitney *U* test, with *P* values indicated for statistically significant differences. Abbreviations: GMT, geometric mean titers; OI, opsonophagocytic activity.

To explore whether differential IgG subclass transfer might explain the poor correlation between binding IgG and function in maternal sera, we measured IgG1-, IgG2-, and IgG3-specific antibodies for O1αβ,2α in a subset of mother and infant pairs (n = 21, restricted by availability of serum volumes). Mothers had higher levels of specific IgG subclasses than their infants, although this only achieved significance for IgG2 and IgG3 (Figure 4). TRs were slightly higher for IgG1 (0.7; 95% CI, 0.55–0.88) compared with IgG2 (0.55; 95% CI, 0.38–0.78) and IgG3 (0.52; 95% CI, 0.34–0.80), but not significantly different than TRs measured for total specific IgG in the healthy controls (Table 2). Correlations between IgG subclasses and functional immunity were generally positive but did not reach statistical significance in maternal or infant sera (Supplementary Figure 3). We measured anti-O1αβ,2α IgM in maternal sera to see if this was present and might be contributing to killing and thus explain the absence of a correlation between maternal IgG and function. While IgM was detectable, specific IgG correlated negatively with function (Supplementary Figure 4).

**Figure 4.**
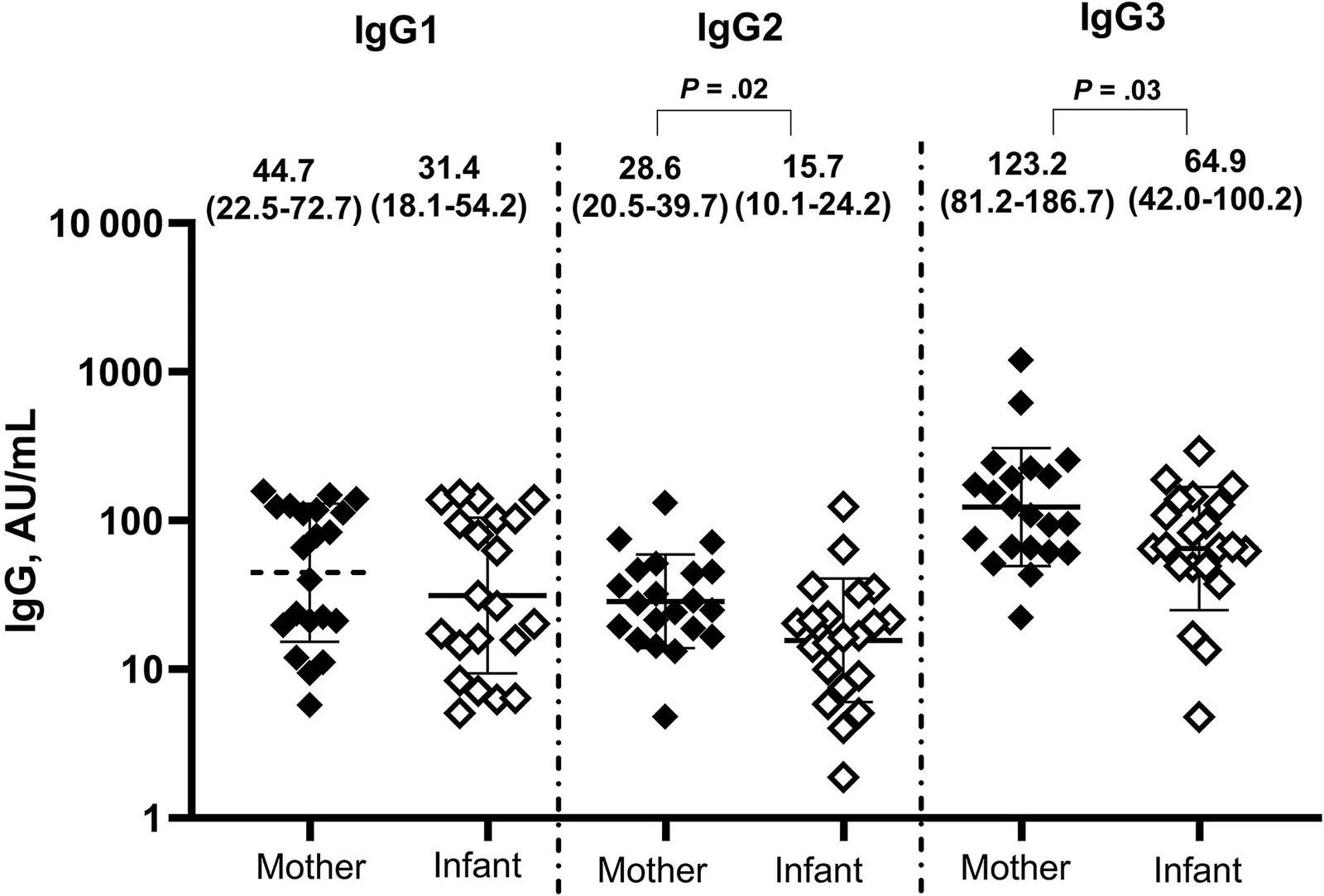
Anti-O1αβ,2α IgG subclass titers in healthy control dyads (n = 21). Geometric mean values are included on the top of each scatter plot. Group comparisons were performed using an unpaired *t* test, with *P* values indicated for statistically significant differences. Infant controls showed lower antibody titers for IgG2 and IgG3 than their mothers. Abbreviation: IgG, immunoglobulin G.

The OIs for mothers of cases (3725) and healthy controls (3250) were similar but significantly lower than those of mothers of sick controls (9890; *P* = .009 and *P* < .0001, respectively). When comparing titers in infants, the OI of sick control infants (180.4) was greater than that of cases (89.4) and healthy control infants (63.08), although only the latter was significant (*P* = .04).

## DISCUSSION

This study investigated naturally acquired IgG to *K. pneumoniae* O1 antigens as well as functional *K. pneumoniae* immunity in mother–infant pairs in Malawi a setting with a high burden of neonatal sepsis. O1 is an O antigen terminating the LPS structure found on ∼50% of strains causing neonatal infection in LMICs and thus an important potential target for inclusion in vaccines designed to protect neonates from *K. pneumoniae* infection [13]. Whether O1-specific antibodies in humans are protective is currently unclear, but if they were shown to be linked to protection, O1 antigen could be included in a vaccine and the measurement of natural O1 immunity might contribute to developing correlates of protection. This approach has been advanced for group B *Streptococcus* (GBS), where thresholds for risk reduction of GBS disease based on natural levels of IgG to capsular polysaccharide in infants have been accepted as an end point for the licensure of a 6-valent GBS capsular polysaccharide glycoconjugate vaccine [22]. While 1 case–control study conducted in Botswana suggested an association between low levels of anti-*Klebsiella* antibodies and susceptibility to *K. pneumoniae* infection, they measured IgG antibody to a bacterial lysate, and thus it is unclear what target antigen was being measured [14].

All of the mothers investigated had similar levels of anti-O1 antibodies and detectable functional immunity in their sera. Anti-O1 IgG was efficiently transferred to their babies, although the titers were highest in control babies due to the older postnatal age of the cases. All of the cases and sick controls had blood samples taken at the time of infection (median, 8 days), by which time antibody had naturally declined. A similar confounding has complicated the evaluation of natural immunity to other pathogens causing infection in early life. These have hampered efforts to recognize the validity of thresholds of protection generated in similar case–control studies [23, 24]. TRs at birth for anti-O1 IgG in our study were similar to those described for *K. pneumoniae*–specific LPS (and *Pseudomonas* and GBS IgG) in a study of twins in Brazil [25], as well as for other pathogen-specific antibodies in the literature [15]. Antibody levels declined rapidly in the postnatal period in this study, a pattern also seen for anti-GBS IgG, where the half-life in infants was estimated to be 27.9 days [26].

Our study was able to generate data on *Klebsiella-*specific functional immunity and demonstrated that levels of functional immunity transferred from mother to infant were significantly lower than antigen-specific IgG TRs. While IgG subclasses may be differentially transported across the placenta and may have different functional activity [27, 28], we demonstrated similar transfer rates for IgG subclasses compared with anti-O1 total IgG. Post-translational modification of IgG, particularly Fc glycosylation, plays a critical role in determining antibody effector function and differs between maternal and neonatal antibodies [29–31], but we were not able to study this due to the limitation of sample volumes.

While binding levels of anti-O1 IgG in control infants correlated well with function, this correlation was not seen in their mothers. O1-specific IgM could account for higher functional immunity in mothers as it does not cross the placenta, but while detectable in maternal serum, it did not correlate with function. A similar discrepancy between the maternal transfer ratio of antigen-specific IgG compared with functional antibodies has been described for *Shigella flexneri* 2a in a study in Malawi. Ndungo and colleagues were, however, able to demonstrate that the enhanced activity against *S. flexneri* 2a in maternal sera was attributable to maternal anti-LPS IgM [32].

Reduced functional immunity in infants compared with mothers as demonstrated in this study may underpin susceptibility to *K. pneumoniae* infection in early life, justifying the exploration of maternal immunization to prevent early-life infection.

In this study, immunity to O1αβ,2α and immunity to O1αβ,2β were highly correlated. We have previously shown that antibodies generated by vaccination of both rabbits and humans with O1α,2α were cross-reactive with O1αβ,2β [20, 33], and recent studies have shown cross-reactive O1αβ,2α- and O1αβ,2β-specific IgG, IgM, and IgA in patients with bloodstream infection [34]. Given that O1αβ,2α and O1αβ,2β account for ∼50% of clinical infections, this cross-reactivity has important implications for vaccine design and suggests that, should anti-O1 antibody be shown to be protective, targeting a single O1 subtype may provide relatively broad vaccine coverage [13].

Our study has several limitations. The number of cases with *K. pneumoniae* infections was lower than expected, and thus the power to detect differences between cases and controls was diminished. The absence of cases in the antenatal cohort led to a discrepancy in the ages of the cases vs the controls that precluded a meaningful analysis of antibody thresholds. In addition, a third of heathy controls were enriched for maternal *K. pneumoniae* stool carriage, which may have influenced immunity levels in infants born to those mothers. Future research should leverage larger, multisite cohorts with longitudinal sampling from birth and better matching of cases and controls to help define risk reduction thresholds. The O1 functional assays used in our study were developed and optimized for vaccine evaluation. As these assays use live *K. pneumoniae* strains (KL3 and KL136 capsule in our laboratory), we cannot rule out that some preexisting IgG to KL3 or KL136, or common proteins, may exist in the sera we studied and thus be responsible for some of the functional activity seen. Protocols for measuring immunity, and in particular functional immunity, will need to be agreed upon, and reagents including purified antigen and recognized control sera and standards will need to be developed to enable comparative data to be generated between laboratories and studies.

In summary, we evaluated natural immunity to *K. pneumoniae* O1 antigens in mothers and infants in Malawi. Antibodies to O1αβ,2α and O1αβ,2β are widely detected in mothers and babies and are functional, but levels in infants are significantly lower than in their mothers. Should the antibody levels demonstrated in this study subsequently prove to be protective, these data may help establish thresholds for risk reduction of infection in neonates that could help guide *K. pneumoniae* vaccine and therapeutic or prophylactic monoclonal antibody development.

## Supplementary Material

ofag676_Supplementary_Data

## Supplementary Data

Supplementary materials are available at *Open Forum Infectious Diseases* online. Consisting of data provided by the authors to benefit the reader, the posted materials are not copyedited and are the sole responsibility of the authors, so questions or comments should be addressed to the corresponding author.
