# Supplementary material for: Natural Immunity to Klebsiella pneumoniae in Mothers and Their Infants in Malawi
Source: Open Forum Infect Dis. 2026 Oct 22;13(11):ofag676. doi: 10.1093/ofid/ofag676 (PMC13736112; doi:10.1093/ofid/ofag676)
Supplement: Supplementary file 1 [file ofag676_supplementary_data.docx]

# **Supplementary Material**

**Methods:**

**Genomic Sequence Analysis for Strain Typing**

Species confirmation was performed using Kraken v1.1.11. Annotated assemblies for the short read data were produced using the pipeline described previously [35, 36]. De novo assembly of genome sequences was performed using SPAdes v3.14.0, trailing different kmer lengths between 41 and 127 to find the optimal kmer length [37]. An assembly improvement step was applied to the assembly with the best N50 and contigs scaffolded using SSPACE v2.0 and sequence gaps filled using GapFiller v1.11 [38, 39]. Assembly statistics were generated using the Sanger Pathogens pipeline as available on GitHub. Samples with <20 or >200 contigs and a genome size of <4.4MB or >5.6MB were excluded. Automated annotation was performed using PROKKA v1.5 and genus specific databases from RefSeq [40]. The improved assembly step was done using a WSI developed software [19].

**Supplementary Table 1. A summary of the organisms cultured from sterile sites in the sick controls**

| **Blood culture or CSF result** | **Nb of sick controls** |
| --- | --- |
| *Acinetobacter baumannii* | 4 |
| *Bacillus* species | 4 |
| *Escherichia coli* | 4 |
| Diphtheroids | 3 |
| *Enterobacter cloacae* | 2 |
| *Enterococcus faecalis* | 2 |
| *Proteus mirabilis* | 1 |
| *Pseudomonas luteola* | 1 |
| *Pseudomonas stutzerii* | 1 |
| *Morganella morgani* | 1 |
| Coagulase negative *Staphylococcus* | 1 |
| No growth | 2 |
| No data available | 13 |

Some samples were positive for more than one organism.

**Supplementary Table 2. The accession numbers, ST, O and K loci of the invasive *K. pneumoniae* identified in this study**

| **Neonatal ID** | **Maternal ID** | **ST** | **O locus** | **K locus** | **Sample accession** |
| --- | --- | --- | --- | --- | --- |
| BBY11653 | MTR11673 | ST985 | O1αβ,2β | KL39 | ERS9756174 |
| BBY10812 | MTR10824 | ST25 | O1αβ,2β | KL2 | ERS9756117 |
| BBY11570 | MTR11616 | ST25 | O1αβ,2β | KL2 | ERS9756178 |
| BBY14244 | MTR14248 | ST261 | O2β | KL38 | ERS9756176 |
| BBY14772 | MTR14768 | ST73 | O1αβ,2α | KL25 | ERS9756193 |
| BBY15258 | MTR15252 | ST39 | O1αβ,2β | KL23 | ERS16233445 |
| BBY15274 | MTR15245 | ST268 | O2α | KL20 | ERS10423831 |
| BBY15829 | MTR15856 | ST39 | O1αβ,2β | KL62 | ERS16233442 |
| BBY15985 | MTR16094 | ST39 | O1αβ,2β | KL62 | ERS10423803 |
| BBY70139 | MTR18124 | ST268 | O2α | KL20 | ERS16233461 |
| BBY20019 | MTR19296 | ST39 | O2β | KL23 | ERS16233576 |
| BBY31370 | MTR20732 | ST607 | O1αβ,2α | KL25 | ERS16233606 |
| BBY31214 | MTR20583 | ST45 | O2α | KL43 | ERS16233471 |
| BBY31040 | MTR20419 | ST292 | O3b | KL38 | ERS16233607 |
| BBY30695 | MTR20062 | ST889 | O1αβ,2α | KL54 | ERS16233450 |
| BBY31669 | MTR21011 | ST874 | O1αβ,2α | KL62 | ERS16400188 |
| BBY32253 | MTR21573 | ST307 | O2β | KL102 | ERS16400203 |

**Supplementary Table 3.** maternal-to-infant transfer for functional activity in cases, healthy and sick controls.

| **Serotype** | **Samples** | **Infant: mother ratio binding activity** | **Infant: mother ratio functional activity** |
| --- | --- | --- | --- |
| O1αβ,2α | Cases | 0.28 | 0.024 |
|  | Healthy controls | 0.67 | 0.02 |
|  | Sick controls | 0.34 | 0.02 |
| O1αβ,2β | Cases | 0.31 | 0.08 |
|  | Healthy controls | 0.63 | 0.13 |
|  | Sick controls | 0.35 | NA |

**Supplementary Figure 1**. Correlation between O1αβ,2α and O1αβ,2β IgG binding (AU/ml) in all mothers (A) and infants (B) and functional immunity in mothers (C) and infants (D).

**Supplementary Figure 2.** Correlation between IgG binding (AU/ml) to O1αβ,2α and O1αβ,2β and functional activity (OI) to O1αβ,2α and O1αβ,2β among a subset of maternal (A) and infant (B) healthy controls

**Supplementary Figure 3**: Correlation between IgG subclasses titres and functional activity in mothers and infants (n=21 dyads).

**Supplementary Figure 4.** Correlation between IgM specific for O1αβ,2α and functional activity against O1αβ,2α in selected mothers (n=21).
